# Origins of Balance Disorders during a Daily Living Movement in Obese: Can Biomechanical Factors Explain Everything?

**DOI:** 10.1371/journal.pone.0060491

**Published:** 2013-04-03

**Authors:** Jean-Baptiste Mignardot, Isabelle Olivier, Emmanuel Promayon, Vincent Nougier

**Affiliations:** UJF-Grenoble 1, CNRS, TIMC-IMAG UMR 5525, Grenoble, France; University of Sao Paulo, Brazil

## Abstract

Obese people suffer from postural deficits and are more subject to falls than their lean counterpart. To improve prevention and post-fall rehabilitation programs, it seems important to better understand the posturo-kinetic disorders in daily life situations by determining the contribution of some key factors, mainly morphological characteristics and physical activity level, in the apparition of these disorders.

Twelve severe android obese and eight healthy non obese adults performed a reaching task mobilizing the whole body. To further determine the origin of the postural and motor behavior differences, non obese individuals also performed an experimental session with additional constraints which simulated some of the obese morphological characteristics. Impact of the sedentary lifestyle was also studied by dissociation of the obese in two subgroups: physically « active » and physically « inactive ». Movement kinetics and kinematics were characterized with an optoelectronic system synchronized to a force platform. The mechanical equilibrium pattern was evaluated through the displacements of the Centre of Mass (CoM) and the centre of foot pressure within the Base of Support (BoS).

Results showed that obesity decreased movement speed (≈−23%, p<0.01), strongly increased CoM displacement (≈+30%, p<0.05) and induced an important spatio-temporal desynchronization (≈+40%, p<0.05) of the focal and postural components of the movement during the transition between the descending and ascending movements.

The role of some morphological characteristics and of physical activity on obese patients' postural control disorder is discussed and set back in the more general context of overall factors contributing to postural deficits with obesity.

## Introduction

Many studies established a link between obesity and posturo-kinetic deficits during upright quiet stance [1]–[5], functional daily living tasks [6]–[9], or gait [10]–[15]. Other studies showed a link between obesity and the risk of falling [16]–[18] or bone fracture [19]–[23]. In order to improve prevention and rehabilitation programs of postural control deficits related to obesity, it seems important to better understand the posturo-kinetic deficits in daily life situations by further determining their origin.

From a biomechanical point of view, postural control during daily living activities is regulated by the horizontal CoM acceleration which is mainly associated to the horizontal distance between CoM and CoP [24]. Human erect posture is slightly tilted forward and the CoM is projected forward of the ankle joint. A healthy adult human body standing upright can be modelled as a system with a single degree of freedom (the ankle), that is, as an inverted pendulum. The orthogonal projection of the top of the pendulum corresponds to the CoM which is located slightly ahead of the base of the pendulum (ankle). This configuration tends to bring down the system forward. To fight against this potential fall, the system must generate forces that will act as shrouds. When CoM is located forward of the ankle, the muscles ensuring plantar flexion must act. Conversely, when CoM is located behind the ankle, it is the muscles ensuring dorsiflexion which must act.

In the absence of muscle contraction, the CoP is projected at the ankle joint and in this configuration, the torque generated between CoM and CoP is used, under the action of gravity, to bring the body forward. The principle of balance control is to cancel the generated torque by continuously readjusting CoP under CoM with an adapted contraction of the triceps surae muscle. Conversely, to initiate a movement, it is necessary to accelerate CoM with respect to CoP by a modulation of the resulting torque at the ankle joint by way of muscular contractions [25], [26].

From a morphological point of view, obesity is characterized by an excessive fat mass accumulation [27]. The distribution of this additional mass is uneven across the body regions, and in obese android patients it is mainly located on the trunk, especially the abdominal area [28], [29]. Therefore, changes of weight distribution modify de facto CoM location of each segment affected by these changes. In other words, to maintain the position of the whole body CoM, the arrangement of body segments relative to each other must change.

From the point of view of the physical lifestyle, obesity is frequently associated to a sedentary behavior and the prevalence of obesity in the physically inactive population is high [30]–[34]. Starting from the premise that each motor situation during daily life contributes to the improvement of motor skills, a sedentary lifestyle may affect the capacity to control posture in obese especially during goal oriented motor tasks requiring an important equilibrium component.

**Figure 1:**
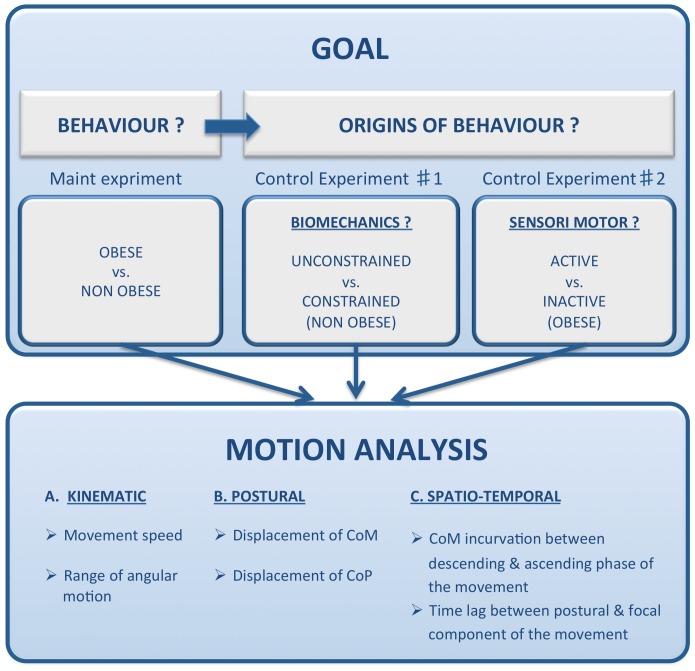
Illustration of the experimental design and framework hypotheses. The differences in posturo-kinetic behaviour (Main experiment) was investigated first; then, the role of some biomechanical (control experiment #1) and sensory-motor (control experiment #2) factors underlying these postural/motor deficits was identified by comparing unconstrained vs. constrained non obese participants and active vs. inactive obese patients, respectively.

The present study compared the motor control behavior of obese to their lean counterpart through the analysis of a Whole Body Reaching (WBR) task, in which subjects were asked to reach an object placed in an outer body area. This task was chosen, on one hand, because it is a situation of everyday life, essential for maintaining subjects' independence, as for example to pick up the keys fallen to the ground. On the other hand, it is a challenging task involving a combination of strong postural (maintaining equilibrium while bending forward) and focal constraints (movement directed towards a target). To reach the target, the subject must mobilize a large part of her/his body segments to execute an appropriate muscular coordination. In a first step, we aimed at determining the kinematics, postural and temporal characteristics of the obese posturo-kinetic behavior by comparing an android obese type II group with a group of non obese participants, (**Figure 1**). Then, we aimed at identifying the role and contribution of some morphological characteristics and of the physical activity lifestyle in the observed posturo-kinetic deficits. To further understand to which extent the morphological constraints were responsible for the observed changes, we compared the behavior of non obese participants with and without a simulated obese morphology. To better understand to which extent the level of physical activity was responsible for the observed changes, we also compared the behavior of the most physically active obese patients with their inactive counterpart. We hypothesized that obese patients exhibit a different spatio-temporal pattern of motor response than control subjects, for performing the WBR task. This motor pattern should be similar for the control subjects simulating obese morphology, emphasizing the contribution of biomechanical factors. On the other hand, active obese subjects may exhibit responses more similar to those of control subjects.

## Methods

### Ethics Statement

Local ethics committee CERNI “Comité d′Ethique pour les Recherches Non Interventionnelles” of the “Pole Grenoble Cognition” specially approved this study (Ref N°: 2012-09-07-4). This study was conducted according to the principles expressed in the Declaration of Helsinki and all participants gave their written informed consent.

### Participants

A total of twenty individuals took part in this investigation. Twelve voluntary severe android obese adults (obese group, age = 47.1±16 years, total body height = 1.67±0.14 m, BMI = 36.6±3.3 kg.m^−2^, seven women and five men) and eight voluntary healthy non obese adults (non obese group, age = 41.6±14.8 years, total body height = 1.72±0.12 m, BMI = 21.4±2 kg.m^−2^, four women and four men. One-way analyses of variance (ANOVA) with a main factor "group" were performed for non obese vs. obese age, total body height, and BMI, respectively. F(1,18) = 0.59, p>0.05, F(1,18) = 0.76, p>0.05 and F(1,18) = 138.78, p<0.001). All participants underwent a complete medical examination and only individuals free from known muscular, neurological, or severe cardiovascular disease took part in the study.

### Task and procedures

**Figure 2:**
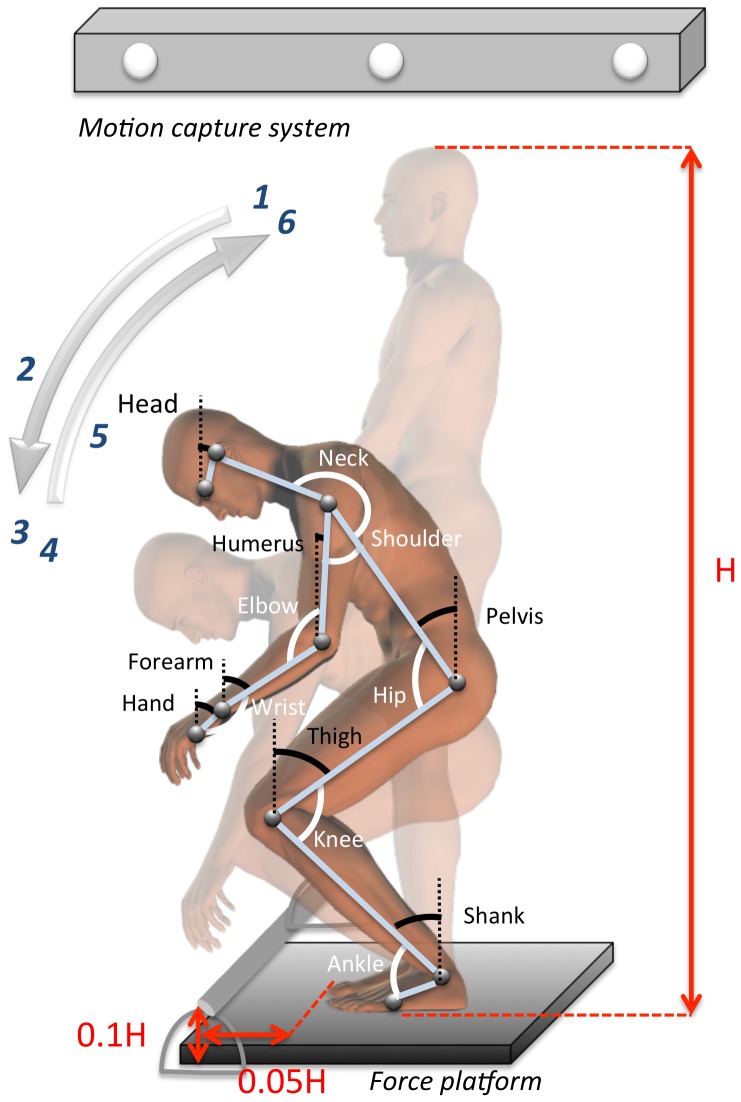
Experimental set-up. Segmental modeling and representation of inter-segmental angles and angles of elevation.

Participants had to perform a WBR task which was divided into two successive components: A descending movement for grasping a bar placed in front of the body followed by an ascending movement for going back to the initial position. To perform this task, participants stood barefoot on a force platform with their feet forming a 30 deg angle relative to each other, with their heels 0.015 m apart. At the start of the recording, they were asked to stand as immobile as possible, with their forearm kept horizontally while staring at a cross target (0.2 m×0.2 m) located 3 m away from the force platform. An auditory signal was randomly triggered 5 to 10 sec after the start of the recording. It signaled the participant to stop staring at the cross target and to initiate the movement toward the bar. As illustrated in **Figure 2**, the aim of the descending movement was to grasp the bar (0.4 m of length, 0.025 m of diameter, 100 g of mass) positioned at a vertical distance from the floor of 10% of the total body height and at a horizontal distance beyond the anterior limit of the BoS of 5% of the total body height. Participants could not take support on the bar because it was placed on a deformable support. While holding the bar, participants were asked to come back to their initial position (ascending movement) without stopping their movement and with maximal stability. The recording was stopped 5 to 10 sec following return to the initial position. No other instructions were given to participants to achieve the movement. In order to modulate the level of complexity of the task, two conditions of execution speed were manipulated: A natural speed (“pick up the bar at a comfortable speed”) and a maximal speed (“pick up the bar as quickly as possible”). To ensure that movement execution was well assimilated, all participants performed few training trials at both speeds. Six trials per speed condition were recorded for analysis. The 12 trials were executed in a random order with a one minute rest between each trial.

The experiment took place in a 6 m×8 m room in which temperature was stabilized with uniform lighting conditions and limited noise disturbances. The motion capture was done via an optoelectronic system (optotrack 3020 ®, NDI, Ontario, Canada) at a frequency of 100 Hz (12-bit A/D conversion) and filtered using a low pass second-order Butterworth filter (10 Hz). Ten active markers were placed on anatomical landmarks as illustrated in **Figure 2**: Eyes (eye), ear (auditory meatus), shoulder (acromion), elbow (ulnar epicondyle), wrist (radial tuberosity), finger (head of the 5th metacarpal bone), hip (greater trochanter), knee (lateral femoral condyle), ankle (lateral malleolus) and foot (fifth metatarsal head). CoP displacements were recorded synchronously with a force platform (OR6-7, AMTI, MA, USA), sampled at 100 Hz (12-bit A/D conversion), and filtered using a low pass second-order Butterworth filter (10 Hz).

### Data analysis

The beginning of the descending movement (t_0_) was determined from the vertical wrist speed, when the instantaneous speed exceeded 5% of its maximal speed. This was a signal derived directly from the focal component of the movement according to the wrist, which was the base of the first distal segment for grasping the target and which exhibited the earliest movement onset among the ten different makers.

The end of the descending movement which was also the beginning of the ascending movement was determined (t_d–a_) when the vertical wrist speed was null. The end of the ascending movement (t_f_) was determined from the vertical wrist speed, when the instantaneous speed was less than 5% of its maximal speed. Three types of dependent variables were assessed: Three kinematics, five postural, and one temporal variable.

The kinematics dependent variables extracted from the displacement of the three-dimensional anatomical markers were: (1) wrist mean speed during the descending movement; (2) range of angular motion of the seven elevation angles, that is, motion of the body segment relative to the vertical gravity. These angles allowed to assess the kinematics of each body segment as for example the trunk inclination with respect to the vertical during the movement. These seven angles modeled on **Figure 2** were: Shank, thigh, pelvis, head, humerus, forearm, and hand; (3) range of angular motion of the seven inter-segmental angles. These angles measured the kinematics of each body joint, for example the hip range of angular motion during the movement. These seven angles modelled on **Figure 2** were: Ankle, knee, hip, neck, shoulder, elbow, and wrist.

The five postural dependent variables were derived from the estimation of CoM location on the basis of Winter's [35] anthropometric table, based on the previous Dempster's data [36] and from CoP displacements. The CoM estimation was adapted for the obese population from individual morphological and mechanical data (see **appendix**). The CoM variables were: (4) vertical CoM range, normalized with respect to the anatomical body height (i.e., the vertical distance between the lateral malleolus and auditory meatus markers); (5) Antero-Posterior (A-P) CoM range, that is, the horizontal CoM range normalized with respect to the anatomical BoS length (i.e., the horizontal distance between the lateral malleolus and fifth metatarsal head markers); (6) CoM pathway curvature for the descending and ascending movements. The stabilometric variables taken into account in the present study were: (7) backward CoP displacement, that is, the distance between the initial mean CoP position (5 sec auditory signal) and the maximal backward CoP position; (8) A-P CoP range, that is, the distance between the maximal backward and maximal forward CoP position. These two CoP variables were normalized with respect to the anatomical BoS.

The temporal dependent variable was (9) CoP time lag, that is, the temporal delay between the end of the descending movement and the time of maximal forward CoP position.

All data are reported as mean values ± standard deviation. One-way analyses of variance (ANOVA) with a main factor "group" were performed for the following dependent variables: Angular joint shift, linear CoM and CoP displacements, and Baecke score. Two-way ANOVAs were performed for movement speed, with the mains factors "group" and "speed", and for COM curvature with the main factors "group" and "direction of the movement" (descending vs. ascending). For these two-way ANOVAs, *Post hoc* analyses were assessed using the Tukey's HSD test, whenever necessary. *P* level of significance was fixed at 0.05.

## Results

### Main Experiment: Characterization of posturo-kinetic obese behavior with respect to controls

#### Kinematics variables

**Figure 3:**
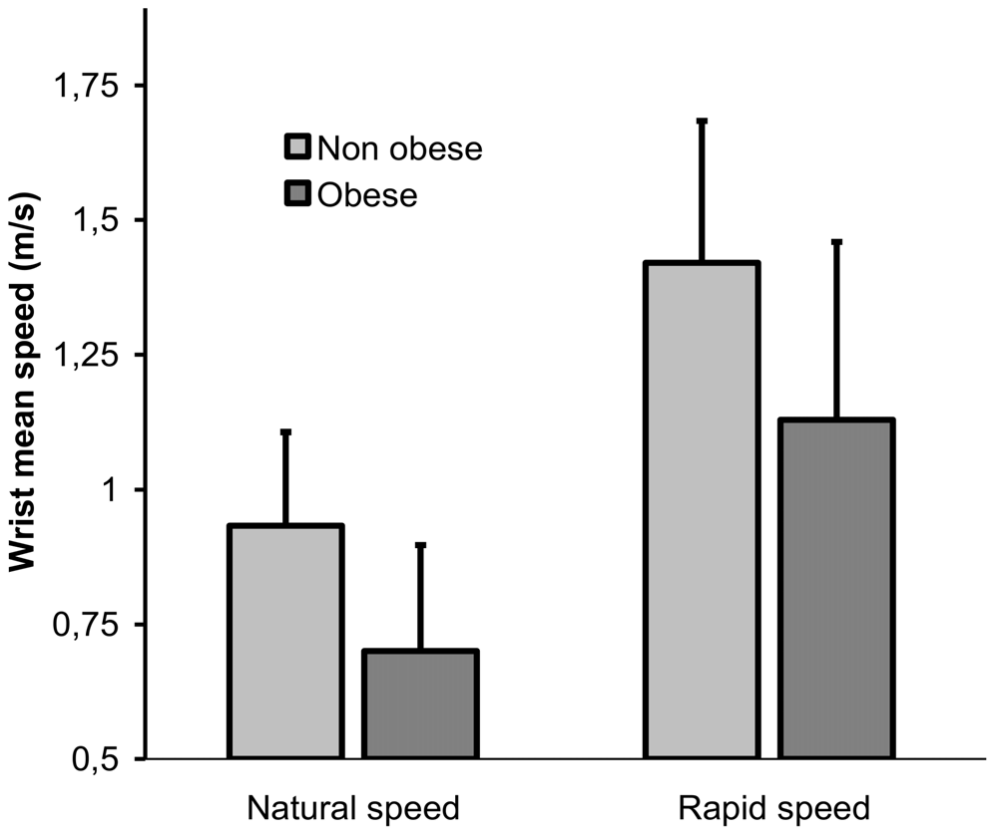
Mean speed of the wrist ± standard deviation during the descending movement for the two groups and the two movement speed conditions.

The wrist mean speed highlighted the velocity at which participants executed the WBR task. Data for the descending movement are summarized in **Figure 3** and **Table 1** The main effect of group (F(1,18) = 5.95, p = 0.025) showed that movement speed was slower for the obese than for the control group (**Figure 3**). As expected, both groups were able to accelerate movement execution in the maximal speed condition as indicated by the significant effect of speed (F(1,18) = 111.57, p = 0.000). There was no significant interaction of group × speed condition. For the other kinematics variables, results did not differ between the two speed conditions. For sake of clarity, only the results for the “natural” speed condition will therefore be further presented.

**Table 1:** Mean values ± standard deviation for the kinematics (wrist speed during the descending movement, elevation and inter-segmental angles), postural (vertical and A-P CoM displacement, total and backward A-P CoP displacement, CoM curvatures of the descending and ascending movements) and temporal (time lag between the end of the descending movement and A-P CoP maximal amplitude) dependent variables, for the two non obese and obese groups.

		Non obese	Obese
Nat sp. Wrist mean sp (m.s^−1^)		0.93±0.17	0.7±0.2
Rap sp. Wrist mean sp (m.s^−1^)		1.42±0.26	1.13±0.33
Elevation angle shift (deg)	Shank	21.92±10.14	16.35±8.5
	Tigh	57.97±9.35	42.66±10.44
	Pelvis	72.5±12.76	75.43±11.19
	Head	54.12±16.31	63.77±15.28
	Humerus	18.16±9.43	13.96±5.48
	Forearm	77.76±10.49	90.61±8
	Hand	61.23±16.05	73.19±13.27
Inter-segmental angle shift (deg)	Ankle	20.09±8.81	17.59±8.49
	Knee	79.93±18.92	58.98±19.31
	Hip	134.9±4.68	116.05±15.86
	Neck	40±10.48	40.73±24.76
	Shoulder	62.51±13.15	65.41±9.34
	Elbow	66.39±12.27	81.15±7.09
	Wrist	16.36±14.34	28.8±17.04
Vert CoM (% of anat BH)		25.89±2.76	22.26±1.88
A-P CoM (% of anat BoS)		78.49±15.58	112.02±39.6
A-P CoP range (% of anat BoS)		110.1±31.94	130.86±33.1
Back CoP (% of anat BoS)		46.83±17.31	24.14±13.64
CoM incurvation Descending (a.u)		0.07±0.04	0.06±0.02
CoM incurvation Ascending (a.u)		0.1±0.06	0.12±0.04
CoP time lag (ms)		−100±70	220±0.430

**Figure 4:**
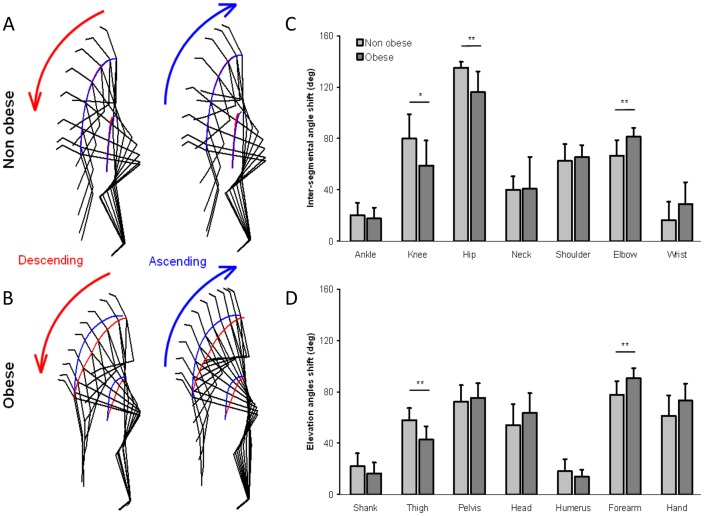
Kinogram representation of the descending and ascending movements executed at a natural speed for one non obese and one obese participant, representative of their respective groups. The CoM and shoulder pathway are shown in red for the descending movement and blue for the ascending movement (a–b). Mean angle range of motion of the joint and mean elevation angle between the start and the end of the descending movement for the two non obese and obese groups (c–d).

Angular variations between the beginning (t_0_) and end of the descending movement are illustrated in **Figures 4c-d** and **Table 1**. A one-way ANOVA was used to examine the effect of group for each angle. The angular range was smaller for the obese than for the non obese participants, for the knee, hip, and thigh (F(1,18) = 5.74, 10.51, 11.18, p = 0.0277, 0.0045 and 0.0036, respectively). In other words, the obese patients exhibited a smaller lower limbs flexion and trunk inclination than their lean counterpart. In contrast, the angular range for the elbow and forearm was greater for the obese than for the non obese participants (F(1,18) = 11.72 and 9.69, p = 0.003 and 0006, respectively). To grasp the bar, the smaller flexion of the lower limbs in the obese patients was compensated for by a larger extension of the upper limbs. The angular amplitude of the other angles (ankle, neck, shoulder, wrist, shank, pelvis, head, humerus and hand) was similar for both groups. The movement kinematics is also represented using kinograms (**Figures 4a–b**) for the descending and ascending movement for a representative trial of non obese and obese individuals. The picture frequency is 10 Hz, such that two static kinograms are separated by 0.1 s. The two-dimensional CoM and shoulder pathways are symbolized by the red and blue paths for the descending and ascending movements, respectively. As can be observed on the kinograms, flexion of the lower limbs and trunk was smaller for the obese patients than for the control group, with a smaller forward knee and backward hip motion.

#### Postural variables

**Figure 5:**
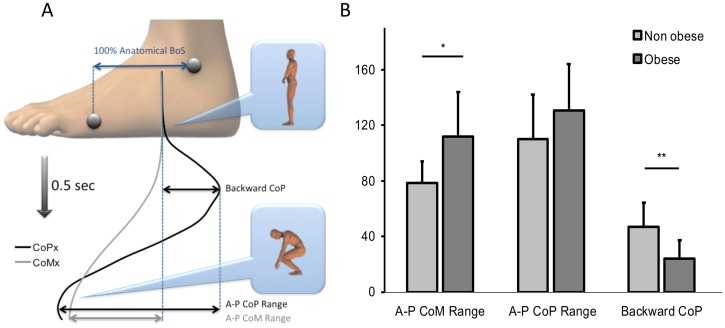
Mechanical equilibrium pattern. A typical pattern for a non obese participant modeled by the horizontal mobility of CoP and CoM into the BoS during the descending movement (a). A-P CoP and CoM range displacement, as a percentage of the anatomical distance from the BoS for the entire (descending + ascending) movement (b).

The equilibrium pattern is illustrated in **Figure 5a** for a representative participant of the control group by the A-P CoM and A-P CoP displacements within the BoS. The vertical axis represents the time elapsed between the start of the movement (upper part of **Figure** 5a) and the end of the descending movement (lower part of **Figure** 5a). As can be seen, there was first a backward CoP displacement necessary to generate the forward torque between CoP and CoM. Then, CoM moved earlier during movement execution until it reached a maximal forward value at the end of the descending movement. Finally, the CoP moved forward too, to reach the same A-P position as CoM in order to (1) cancel the forward torque, (2) reverse CoM direction of rotation with respect to the ankle joint and (3) initiate the ascending movement. A one way ANOVA examined the effect of group on backward CoP displacement, A-P CoP range, and A-P CoM range. As can be seen on **Figure 5b** and **Table 1**, the backward CoP displacement was smaller in obese patients than in non obese participants (F(1,18) = 11.21, p = 0.004), whereas there was no effect of group on A-P CoP range (F(1,18) = 1.94, p = 0.18). However, A-P CoM range was larger in obese patients than in non obese participants (F(1,18) = 5.13, p = 0.036). The body bending can be assessed with the Vertical CoM variable (Table 1 and Figures 4a–b). A one way ANOVA showed that vertical CoM displacement was smaller for the obese than for the non obese group (F(1,18) = 12.3, p = 0.0025).

**Figure 6:**
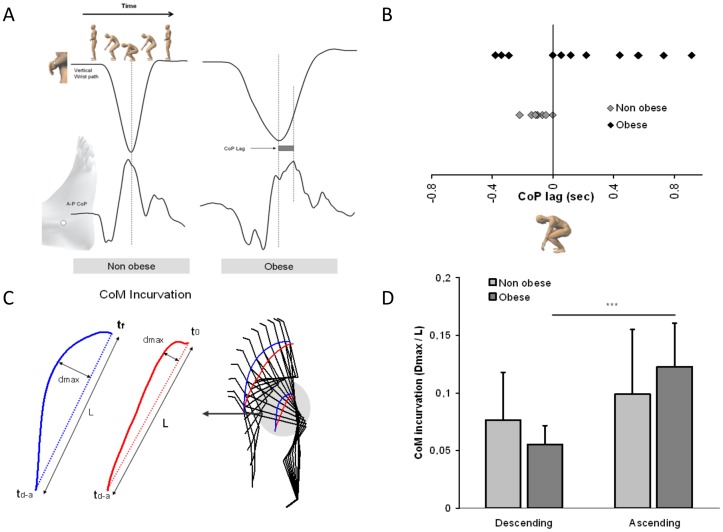
Spatio-temporal synchronization of the focal and postural components of the movement during the transition between the descending and ascending movements. The time lag between the end of the descending movement (i.e vertical wrist pathway) and the forward CoP peak (a). This difference was quantified and the average score for each individual of the two groups is shown in (b). The curvature profile of the CoM trajectory was calculated for the descending and ascending movements by the d_max_/L ratio (c). Results for the two groups and the two movement directions are shown in (d).

Observation of the CoM and shoulder pathways (**Figure 4a–b**) showed symmetry between the descending and ascending movements for the non obese participants whereas for the obese patients the ascending path of the movement strongly differed from that of the descending movement. This trajectory shift was quantified by CoM curvature (**Figure 6c**). A 2 groups × 2 two movement directions ANOVA showed a significant main effect of movement direction (F(1,36) = 13.36, p = 0.0008) and a significant interaction of group × movement direction (F(1,36) = 3.26, p = 0.0079, **Figure 6d** **,** **Table 1**). The post hoc analysis showed that the curvature of the descending and ascending movements was similar for the non obese individuals, whereas for the obese group the curvature of the ascending movement was twice the one observed during the descending movement.

#### Temporal variable

In addition, there was a consistent time lag between the end of the descending movement (i.e., when wrist vertical speed was null) and the maximal forward CoP position for the obese individuals. In contrast, the maximal forward CoP position was well synchronized with the end of the descending movement for the non obese individuals. The CoP time lag is illustrated in **Figure 6a** for two representative non obese and obese patients. Whereas for the non obese individuals, the maximal forward CoP position occurred between 0 to 220 ms before the end of the descending movement, this maximal CoP position was observed between 55 to 915 ms after the beginning of the ascending movement for 8 out of the 12 obese patients (**Figure 6b** and **Table 1**).

Overall, results of the present experiment showed that obese patients exhibited a different posturo-kinetic behavior than healthy participants, when facing a complex motor task also involving equilibrium. The goal of the two following control experiments was to further identify and isolate some of the factors which were at the origin of the observed differences.

In Control Experiment 1, to assess the role of some morphological constraints on obese postural deficits, six individuals from the non obese group voluntarily participated to a second experimental session following the same experimental procedure but in which the obese morphological characteristics, that is some biomechanical constraints, were artificially engineered. These manipulations aimed at simulating the obese biomechanical constraints to investigate their presumed effects on the posturo-kinetic behavior.

Apart from obvious morphological changes, obesity also indirectly leads to a sedentary lifestyle which may also affect the posturo-kinetic behavior. In Control Experiment 2, to assess the role of regular physical activity on obese behavior, the physical lifestyle of the obese group was also investigated. Using a questionnaire assessing the level of daily physical activity, the twelve obese participants were divided into two physically “active” and physically “inactive” subgroups which were then compared.

### Control Experiment 1: The role of biomechanical constraints induced by obese morphology on posturo-kinetic behavior

**Figure 7:**
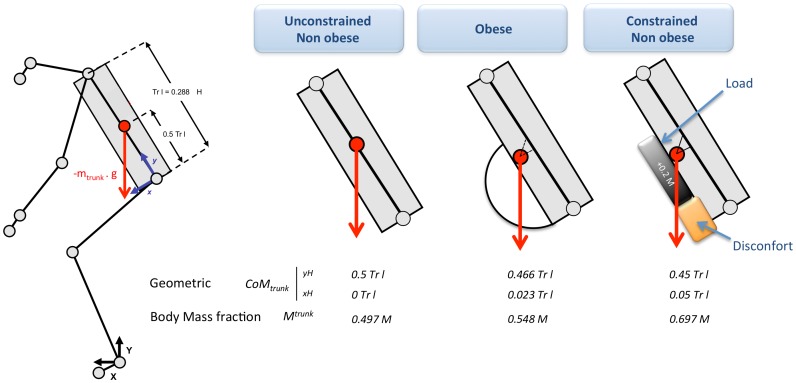
Anthropometric modeled characteristics of the trunk adjusted from the Winter's anthropometric table [**57**]. The morphological and mechanical constraints specific to the obese and non obese participants are modeled by: The coordinates of the CoM trunk into the coordinate system (H, x, y) and expressed as a ratio of the length of the trunk, and the trunk body mass fraction. “Discomfort + load” corresponded to the addition of a morphological and mechanical constraints, which were applied to the non obese participants (Control Experiment 1).

Six individuals from the non obese group were asked to perform the same WBR task whereas some physical constraints were engineered. A slightly deformable rigid foam cube, without mass (<10 g), was fixed at the level of participants' pelvis in order to simulate the discomfort due to an excess of abdominal fat. An additional charge (20% of initial body weight) was loaded at the abdominal level in order to simulate the mechanical properties of the excess of mass on the trunk segment. The additional charge was distributed evenly in a rucksack worn on the abdomen from the manubrium sternal joint to the navel. The pressure caused by carrying the rucksack was distributed over the shoulders and different trunk areas since the shoulders straps were also attached together in the participants' back at the level of the L8/L9 vertebrae (**Figure 7**). This constraint increased the body mass fraction of the trunk and displaced the location of the trunk CoM (see **appendix**). These manipulations aimed at simulating the major biomechanical constraints (discomfort and overload) endured by obese patients during the WBR task. Within the context of this dynamic motor task mobilizing the whole body, a prerequisite for providing a good simulation of obese morphology in non obese participants is to find the best compromise between a good quantity/weight distribution and a reduced additional discomfort for executing the movement. Indeed, it is important to minimize all factors that are not directly related to the constraints of obesity, which could interfere with a natural realization of the movement. For example, the compression due to the attachment of the additional masses on the body could stimulate cutaneous receptors that can disrupt the subject. Thus, the trunk only, which is the heavier segment and which was strongly mobilized during the WBR task, was overloaded. The compromise mentioned above, even though not perfect, was characterized for the non-obese overloaded group by a lower IMC value (26.8±1.3 kg.m^−2^) but a greater trunk mass fraction than for the obese group, as described in the appendix.

As for the main experiment, after a few practice trials for each movement speed condition, four trials per condition were recorded for analysis. Eight trials in all were executed in a random order with a one minute rest between each trial. The same variables as before were analyzed.

#### Results (Control Experiment 1)

**Figure 8:**
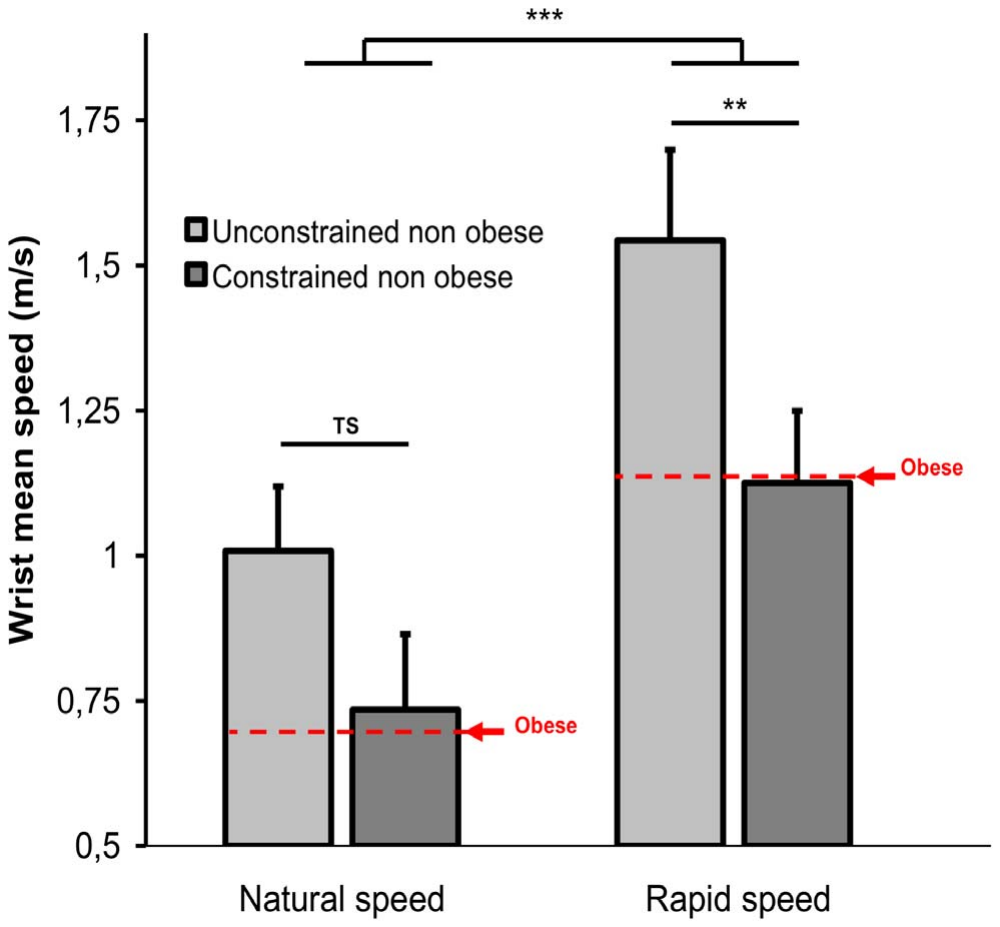
Mean speed of the wrist ± standard deviation during the descending movement for the non obese group with and without additional biomechanical constraints in the two speed conditions.

A 2 groups (unconstrained vs. constrained) × 2 speed conditions ANOVA with repeated measures was applied to movement speed. As for the obese vs. non obese comparison, movement speed was slower for the constrained than for the unconstrained non obese group (F(1,10) = 25.36, p = 0.0005, 0.93 vs. 1.28 m.s^−1^). As shown by the main effect of speed, both groups were able to accelerate movement execution (F(1,10) = 206.13, p<0.001, 0.73 vs. 1.13 m.s^−1^ and 1.01 vs. 1.54 m.s^−1^, for the constrained and unconstrained non obese groups, respectively, **Figure 8**, **Table 2**). There was also a significant interaction of group × speed (F(1,10) = 5.06, p = 0.048) which post hoc effects are illustrated in **Figure 8**. The effect of execution speed was greater for the constrained than for the unconstrained non obese participants. For the two other kinematics variables (elevation and inter-segmental angles) no significant difference was observed.

**Table 2:** Mean wrist speed ± standard deviation for the natural and maximal speed conditions for the non obese group without (unconstrained) and with (constrained) biomechanical constraints and for the two obese active and inactive subgroups.

		Control Experiment 1		Control Experiment 2	
		Unconstrained	Constrained	Active obese	Inactive obese
Nat sp. Wrist mean sp (m.s^−1^)		1.01±0.11	0.73±0.13	0.84±0.13	0.56±0.16
Rap sp. Wrist mean sp (m.s^−1^)		1.54±0.16	1.13±0.12	1.4±0.18	0.86±0.18
Elevation angle shift (deg)	Shank	22.33±10.33	23.17±12.70	19.54±10.71	13.17±4.48
	Tigh	57.93±7.93	49.5±8.63	46.72±12.56	38.59±6.5
	Pelvis	62.62±11.02	71.65±12.92	71.56±12.91	79.31±8.55
	Head	37.28±11.24	46.85±12.10	68.03±17.57	58.44±11.95
	Humerus	14.72±7.64	5.91±2.67	12.85±5.80	15.07±5.42
	Forearm	80±7.73	84.35±7.74	90.26±9.04	90.96±7.67
	Hand	73.10±19.16	84.20±8.85	74.89±7.87	71.78±17.24
Inter-segmental angle shift (deg)	Ankle	18.54±5.55	19.39±7.09	20.23±8.93	12.3±6.1
	Knee	78.86±15.9	75.76±9.19	68.52±21.07	49.44±12.58
	Hip	131.26±6.45	128.25±3.63	122.92±7.29	109.19±19.7
	Neck	40.48±10.61	43.18±31.39	38.42±27.93	42.66±24.33
	Shoulder	67.81±14.27	68.18±10.93	61.57±9.87	69.25±7.70
	Elbow	75.39±18.36	80.38±12.56	79.68±5.38	82.61±8.75
	Wrist	16.75±14.69	16.69±12.90	31.58±24.41	26.02±7.83
Vert CoM (% of anat BH)		25.50±0.6	25.05±0.56	22.75±1.71	21.77±2.08
A-P CoM (% of anat BoS)		68.38±37.57	105.37±25.91	123.8±33.32	100.24±44.81
A-P CoP range (% of anat BoS)		97.83±34.31	123.48±28.57	131.2±32.02	130.49±37.22
Back CoP (% of anat BoS)		33.63±14.69	26.44±12.16	23.21±14.63	25.07±12.57
CoM incurvation Descending (a.u)		0.035±0.013	0.063±0.001	0.06±0.02	0.05±0.02
CoM incurvation Ascending (a.u)		0.057±0.021	0.081±0.036	0.12±0.04	0.12±0.04

The other results (angle shift, CoM and CoP displacement, and CoM incurvation) are presented only for the natural speed condition.

**Figure 9:**
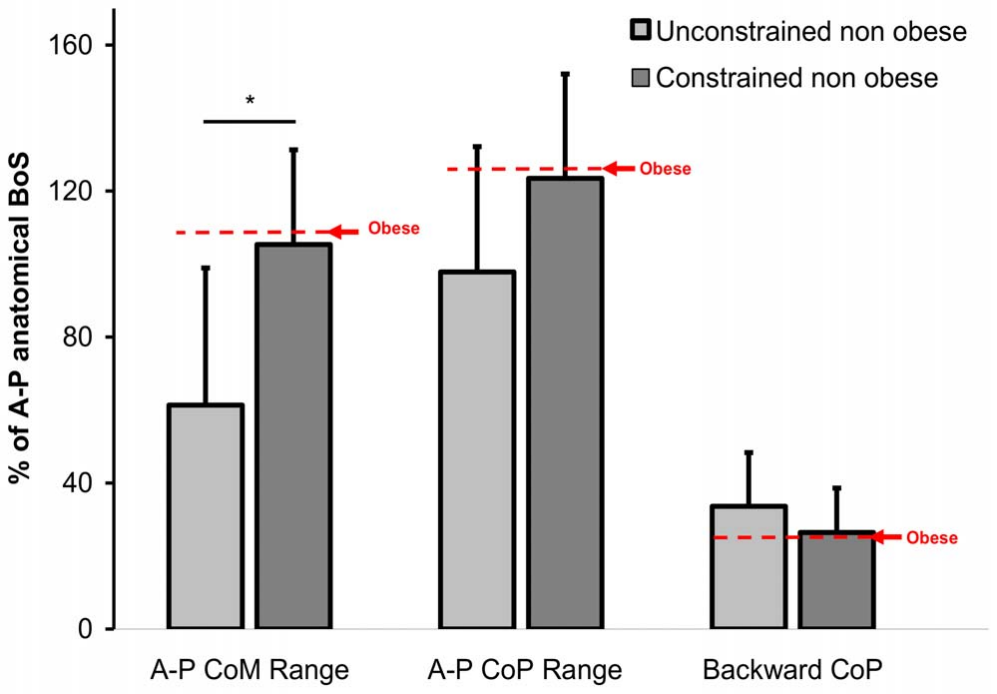
A-P CoP and CoM range displacement, as a percentage of the anatomical distance from the BoS for the descending + ascending movement for the non obese group with and without additional biomechanical constraints.

A one way ANOVA (unconstrained vs. constrained) examined the effect of the morphological constraints on the five postural variables and showed that the mechanical constraints strongly affected A-P CoM range (F(1,10) = 5.57, p = 0.04, 105.37 vs. 68.38% of anatomical BoS for the constrained and unconstrained non obese groups, respectively, **Figure 9**, **Table 2**). Vertical CoM displacement, CoM curvature pathway, A-P CoP range and backward CoP displacement did not statistically differ between the constrained and unconstrained non obese groups. However, backward CoP displacement and A-P CoP range in the constrained condition were close to the values reported for the obese group, as illustrated in **Figure 9** by the red arrows.

There was also no effect of the morphological constraints on the temporal synchronization between the end of the descending movement and the maximal forward CoP position, and on the spatial CoM pathway.

### Control Experiment 2: The role of physical activity on obese posturo-kinetic behavior

To determine the influence of physical activity on the obese posturo-kinetic behavior, the obese group was divided into two subgroups. It is known that the level of psychomotor skills, which are integrated in high-level neural processes, is highly correlated with physically active or sedentary lifestyles [37]–[39]. Thus, the level of regular physical activity of each obese participant was evaluated to categorize them as “active” or “inactive”. The “Baecke questionnaire” [40] was used to assess the level of regular physical activity through three main index scores: (1) physical activity at work, (2) sport practice during leisure time, and (3) physical activity during leisure time excluding sport. Index scores (1) and (3) can vary from 1 to 5 and index score (2) from 0.5 to 5. The sum of these three scores gives a global score which can vary from 2.5 (totally inactive life) to 15 (maximal activity value for each index). Each obese patient was categorized depending on her/his global score. The six participants with the highest scores were included in the “active” subgroup whereas the six participants with the lowest scores were included in the “inactive” subgroup (active obese group; Baecke score = 8.22±0.48, four women and two men, age = 38.33±10.59 years, BMI = 38.32±4.83 Kg.m^−2^; Inactive obese group: Baecke score = 6.21±1.2, three women and three men, age = 55.83±16.4 years, BMI = 35.28±1.25 Kg.m^−2^, F(1,10) = 14.62, p<0.01, 4.82, p>0.05, 4.25, p>0.05 for the active vs. inactive obese Baecke score, age and BMI, respectively).

#### Results (Control Experiment 2)

**Figure 10:**
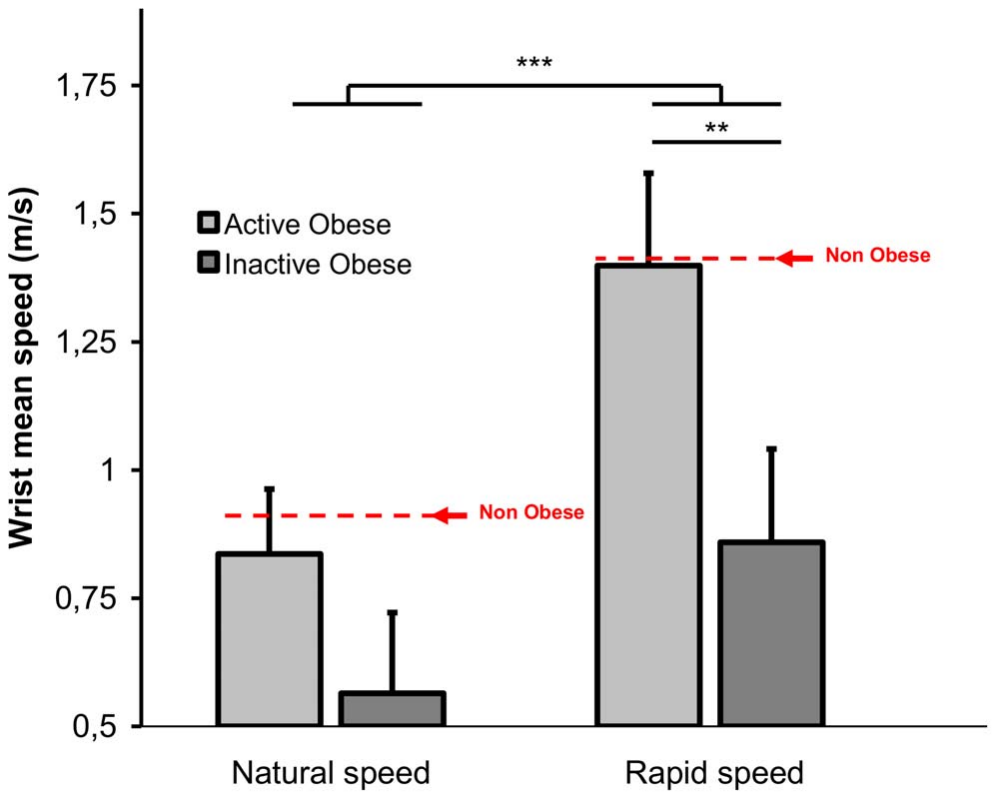
Mean speed of the wrist ± standard deviation during the descending movement for the two active and inactive obese subgroups in the two speed conditions.

A 2 groups (active vs. inactive obese) × 2 speed conditions ANOVA was applied to the wrist speed. As for the obese vs. non obese comparison, movement speed was slower for the inactive than for the active obese group (F(1,10) = 22.24, p = 0.0008, 0.71 vs. 1.12 m.s^−1^). As expected, both groups were able to accelerate movement execution as indicated by main effect of speed (F(1,10) = 123.5, p = 0.000, 0.56 vs. 0.86 m.s^−1^ and 0.84 vs. 1.4 m.s^−1^ for the inactive and active obese groups, respectively, see **Figure 10**, **Table 2**). There was also a significant interaction of group × speed (F(1,10) = 12.08, p = 0.006) which post hoc effects are represented in **Figure 10**. The effect of speed was greater for the inactive than for the active obese patients. The other kinematics, postural and temporal dependent variables were not affected by the level of physical activity.

## General Discussion

The aim of the present study was to investigate the posturo-kinetic behavior of android obese patients and to determine some of the factors underlying the observed deficits (e.g., morphological characteristics and level of physical activity). For this purpose, a WBR task was used as a model of the activities of daily living which present risks of fall.

During dynamic motor tasks such as walking, transfers of postures, or pointing toward targets while standing upright, many authors have shown that obese people perform the motor tasks more slowly than non obese ones [6], [7], [10]–[15]. The originality of the WBR task is to combine focal (target achievement) and equilibration components (maintaining the projection of the CoM inside the BoS) by mobilizing the whole body. Analysis of the mean of the wrist speed (**Figure 3**, **Table 1**) confirmed the slowdown in the obese population, as mentioned previously. However, similarly to non obese people, the obese patients were able to increase their movement speed when required and this speed increase was comparable. It seems clear that the slowdown of the movement observed in obese patients, as compared to non obese individuals of the same age highlighted adjustments of the motor system in response to physiological, morphological, and/or cognitive changes generated by obesity. The kinetics and kinematics movement parameters analyzed in the present study aimed at characterizing these adjustments and at providing some answers regarding the origins of the posturo-kinetic deficits observed in obese patients.

### Angular joint and CoM mobility

For the non obese participants, the angular range of motion and force production capabilities gave them more options in the choice of combined flexion strategies [41]. Contrary to what was thought over many years, healthy people did not seek to stabilize the A-P CoM displacement. Instead, they moved forward their CoM during the WBR task to better control muscular activity at the ankle joint to reduce postural disturbances [41], [42]. This strategy was used even though the option to stabilize the A-P CoM displacement was biomechanically possible [41], [43]. However, the mobilization of A-P CoM during the movement requires generating significant muscle strength.

Considering the relative alteration of muscular strength with obesity [44] and the heavier mass applied to the whole body CoM, which imposed to generate a higher muscular torque to return to a balance state following a disturbance [17], an “obese strategy” aiming at minimizing the A-P displacement of the CoM during the movement was expected. However, the present results did not confirm this hypothesis. On one hand, the flexion amplitude of the lower limb joints (knee, hip and thigh) was smaller in the obese than non obese participants with a decrease of the vertical CoM range. On the other hand, CoM moved more forward in the obese than non obese participants (**Figure 4a–b** and **5b**, **Table 1**). This CoM displacement to the anterior limits of the BoS constituted an important unsafe drop. The elevation angle of the trunk (represented by the pelvis angle) did not differ between obese and non obese participants, indicating that the inclination of the trunk between the beginning and end of the descending movement was similar in both groups. However, the higher trunk body mass fraction of obese patients (see **appendix** and **Figure 7**) increased the forward CoM displacement for a similar trunk inclination. The higher body mass fraction of the trunk and the forwarded CoM_trunk_ location in obese patients also required to generate higher forces during movement execution for a same body geometrical inclination. To reduce force production, especially in the knee extensor muscles, obese patients limited the vertical lowering of CoM as well as the knee joint flexion. Indeed, whereas the maximal voluntary force of knee extensors has been shown to be similar or higher in obese versus non obese people, this force appeared to be lower when normalized to body weight [44], [45]. We also believe that the functional limitations caused by the trunk morphology (e.g., the discomfort induced by the excess of fat) did not allow as wide a range of motion as for non obese participants. This might also explain the reduction in hip flexion observed in the obese patients.

Obese patients must adopt a posture specifically adapted to their morphological characteristics (trunk body mass fraction, hip fat pad discomfort, relative maximal voluntary muscular contraction). However, this body posture was clearly risky because it imposed a significant forward CoM displacement, exposing the obese patients to a higher risk of falling during movement execution [17].

### Spatio-temporal desynchronization between movement kinematics and mechanical equilibrium pattern

To initiate a movement such as the WBR task, it is necessary to generate a torque in particular by a backward CoP displacement, that is, by inhibiting the plantar flexor muscles and/or activating the dorsal flexor muscles. Conversely, the equilibrium principle requires canceling the torque by nullifying the horizontal distance between the CoP and the CoM. In the WBR task, participants had first to initiate the movement to get the object (focal component) while maintaining the projection of the CoM inside the BoS, and then to cancel and reverse the torque at the end of the descending movement to initiate the ascending movement (equilibrium component). As illustrated in **Figure 6b** for the non obese participants, the forward CoP peak was reached shortly before the end of the descending movement. This highlighted the correct time synchronization between movement kinematics and equilibrium pattern. Conversely, for the obese patients there was a time lag between these two events. At this temporal desynchronization was associated the spatial asymmetry of the CoM pathway during the descending and ascending movements. While the curvature of the trajectory remained unchanged between the descending and ascending movements for the non obese group (**Figures 4a–b**, **6c–d** and **Table 1**), the curvature was twice larger for the obese patients during the ascending movement (**Figure 4a–b**, **6c–d** and **Table 1**). In addition, for the non obese participants space-time synchronization was observed between the descending and ascending movements whereas obese patients behave differently suggesting that dynamic postural control was complex and dangerous for the obese patients' physical integrity.

### Factors underlying obese posturo-kinetic deficits: morphological characteristics and physical activity

In order to further quantify the contribution of some morphological characteristics to the obese posturo-kinetic behavior, six subjects from the non obese group performed the same experiment but with additional constraints applied onto the trunk to reproduce the main biomechanical characteristics of obese patients (Control Experiment 1).

The simulation of obese morphological characteristics slowed movement execution (**Figure 8**, **Table 2**). As illustrated in **Figure 8** (see red lines), movement speed was similar for the obese patients and non obese participants disturbed by these artificial morphological constraints, suggesting that morphological factors were directly involved in slowing movement execution. In other words, the morphological discomfort generated by a fat pad disrupting hip flexion combined with the larger body mass fraction of the trunk accounted for the decrease of movement velocity in obese patients.

As previously mentioned, the higher obese trunk body mass fraction (see **appendix**, and **Figure 7**) increased the CoM horizontal movement in the obese participants compared to the same trunk inclination for the non obese participants. The additional results from the constrained and unconstrained non obese participants (**Figure 9**, **Table 2**) confirmed the hypothesis that morphological trunk characteristics contribute to these larger CoM displacements. As illustrated in **Figure 9** and **Table 2**, the A-P CoM range for the non obese participants was larger in the constrained than unconstrained condition. It is likely that the large trunk body mass fraction brought the CoM displacement forward to the BoS boundaries and increased the risk of falling [17].

However, results of Control Experiment 1 were not a simple replication of the main experiment results. For some variables such as joint angle shift, CoM incurvation, and CoP time lag, the constrained non obese behavior was different from the one of the obese patients. Two hypotheses may explain this finding. According to the first hypothesis, the simulated model of obese morphology did not reproduce exactly all the biomechanical characteristics of a real obese body. According to the second hypothesis, the time of application of the mechanical constraints was temporary whereas obese patients constantly live with their overweight. Pregnancy is probably the best simulation model of the morphological trunk characteristics of obese people, even though for a relatively short period. Butler et al. [46], have shown that postural stability is impaired during pregnancy but only from the time in which the morphological changes on the trunk become significant, approximately from the second trimester of pregnancy. This degradation is linear and persists after delivery until 6–8 weeks postpartum, despite the return to a normal body weight. The fact that postural stability of pregnant women was similarly impaired following weight intake and after return to a normal weight clearly suggested that alteration of the implicit sensory-motor knowledge of the body (body schema) rather than biomechanical constraints was involved. However, this interpretation must take into account that postural stability of pregnant women was studied during an orthostatic posture which did not significantly mobilize body segments. During a dynamic task such as the WBR task, it is likely that the abnormal obese posturo-kinetic behaviour resulted from a combination of biomechanical constraints relative to body weight change and an alteration of the body schema.

Independently of BMI factor, it is probable that the quantity of daily physical activity contributes to change the posturo-kinetic skill during WBR tasks. It is well known that the prevalence of obesity in the population of physically inactive people is important [47]–[49]. Therefore, comparison of obese patients' behavior when dissociated into active and inactive obese subgroups allowed investigating this factor.

In addition to the morphological characteristics of the body, sedentary lifestyle also contributed heavily to slow down movement execution in obese patients (**Figure 10**, **Table 2**). This slow down strongly increased with physical inactivity and could be both a consequence of the deficit of muscular strength characterizing obese patients, especially the most sedentary ones, and of the precariousness of the central control of posture. In addition to weight loss and to more classical psychological and physiological effects [50]–[52], regular physical activity could also improve the body schema, suggesting that the movement can be used as a kind of therapeutic tool, helping to improve motor control, especially in pathological populations such as obese patients [53].

From a neurophysiological point of view, for reaching the goals of a postural or motor task, muscular activity must be tightly regulated. The efficiency of this regulation depends on the dialogue between the efferent motor command and the afferent sensory perception of the movement, that is, on the internal models for action [54]. The movement actually achieved is continuously compared with the desired movement (a copy of the efferent motor command) in the intermediate cerebellar cortex. If the comparison reveals an error between the desired state and the real executed state, adjustments can be made to the efferent command.

Repetition and variety of postural and motor situations experienced in daily life, improve the body schema used by the internal models to prepare the movement and make corrections. Accuracy of the initial movement and efficiency of the online corrections are dependent on (1) the wealth of the body schema, built on the previous sensory-motor experience, and (2) the accuracy of the information from the sensory system provided to the high level nervous centers. Accordingly, a sedentary behavior which characterizes most of the obese people would quantitatively and qualitatively alter the body schema.

More generally, the biomechanical constraints and physical lifestyle seemed to be important but not exclusive factors for explaining obese patients' behavior. Some results from the main experiment such as CoM incurvation, CoP time lag, some of the angular shifts, or backward CoP displacement (**Figures 4b,c**, **5b**, **6**, **9**, **Tables 1**, **2**) were not replicated in the two control experiments, suggesting that other factors could explain the deficits observed in obese patients.

### Other obese characteristics may alter postural and motor skills

As some authors have previously demonstrated, information from some sensory receptors in obese patients could be erratic and have a negative impact on postural control [3], [13], [55], [56]. The suppression of visual information induced a greater alteration of the postural performance in obese than in normal-weighted individuals. This suggested that sensory information integrated at a supra spinal level have an accentuated role in maintaining balance for obese patients.

Results from Buschbacher's study [57] showed some alterations in sensory and motor nerve impulses. Sensory and/or motor nerve amplitude is correlated significantly with BMI for median, ulnar, peroneal, and tibial nerves, i.e., and is approximately 20–40% lower in obese than in normal weight subjects. The recruitment of motor units also seems to be altered with obesity [4. These authors suggested that reduced motor units activation and a lower strength per mass ratio are probably important factors contributing to the degraded poorer motor performances of the obese people.

## Conclusion

The present study tried to improve knowledge of the mechanisms behind the functional balance deficits observed in obese patients. The obese patients exhibited a slow movement speed during a reaching task mobilizing the whole body. Associated to this velocity decrease, changes in the kinematics and equilibrium behavior contributed to increase the risk of balance loss. These disorders resulted from a combination of morphological characteristics and physically active lifestyle. However, some of the present results cannot be explained only by morphological characteristics and/or by active lifestyle factors: Additional alteration of neurophysiological, physiological, psychological, and sensori-motor characteristics specific to this pathology must be determined more precisely in future studies.

## Supporting Information

Figure S1 **CoM location in obese.** Modeling of the main anthropometric data from Winter's table (a). Copy of software screenshot which can individually estimate the CoM position of the trunk segment by using a profile photograph of the trunk (b). A statistical comparison of this adjustment method with the conventional method (without adjustment) was performed to control the benefits of the present appendix work. We compared anteroposterior and vertical CoM displacement during the descending movement, analyzed with these two methods and for six trials from six obese participants, randomly selected. The statistical analysis revealed a significant difference between the two methods. For the A-P CoM displacement (% of anat BoS), the result was 132.9±30 vs. 154.4±3.27 without and with adjustment, respectively, t test, t = −4.07, p = 0.0096). For the vertical CoM displacement (% of anat BH), the result was (26.6±3.1 vs. 28.63±3.4 without and with adjustment, respectively, t test = −2.78, p = 0.039). The six trials used for this comparison are illustrated in “c”. As can be seen, there was a temporal difference for the beginning and end of the CoM displacement, depending on the method used.
(TIF)

Text S1 **CoM location in obese: using an Additional Mass Index and picture segmentation to adjust Winter's anthropometric table.**
(DOCX)
