# Supplementary figures and images for: Origins of Balance Disorders during a Daily Living Movement in Obese: Can Biomechanical Factors Explain Everything?
Source: PLoS One. 2013 Apr 3;8(4):e60491. doi: 10.1371/journal.pone.0060491 (PMC3616143; doi:10.1371/journal.pone.0060491)

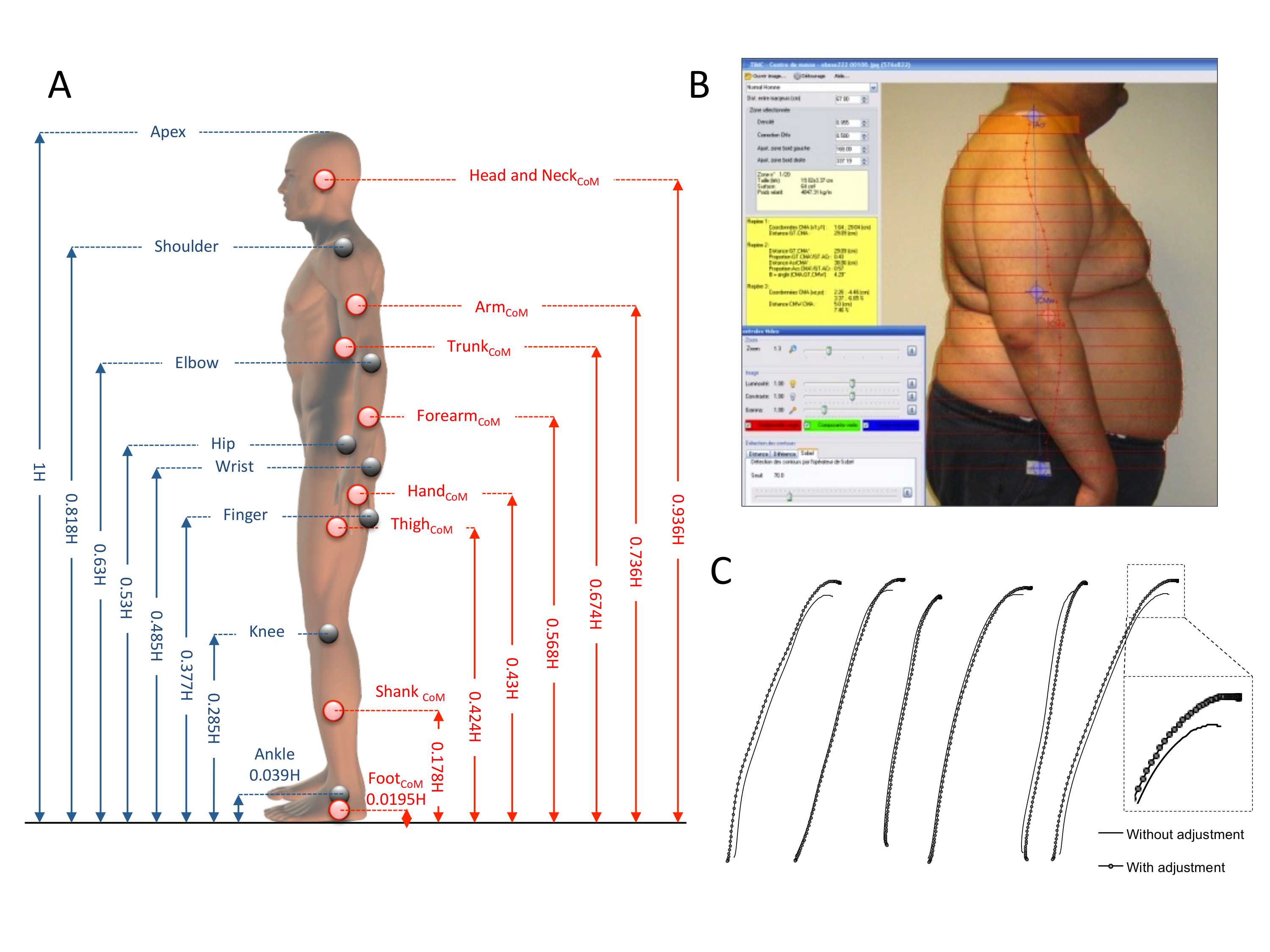

Supplement: Figure S1 — CoM location in obese. Modeling of the main anthropometric data from Winter's table (a). Copy of software screenshot which can individually estimate the CoM position of the trunk segment by using a profile photograph of the trunk (b). A statistical comparison of this adjustment method with the conventional method (without adjustment) was performed to control the benefits of the present appendix work. We compared anteroposterior and vertical CoM displacement during the descending movement, analyzed with these two methods and for six trials from six obese participants, randomly selected. The statistical analysis revealed a significant difference between the two methods. For the A-P CoM displacement (% of anat BoS), the result was 132.9±30 vs. 154.4±3.27 without and with adjustment, respectively, t test, t = −4.07, p = 0.0096). For the vertical CoM displacement (% of anat BH), the result was (26.6±3.1 vs. 28.63±3.4 without and with adjustment, respectively, t test = −2.78, p = 0.039). The six trials used for this comparison are illustrated in “c”. As can be seen, there was a temporal difference for the beginning and end of the CoM displacement, depending on the method used. (TIF) [file pone.0060491.s001.tif]
