# Supplementary material for: Origins of Balance Disorders during a Daily Living Movement in Obese: Can Biomechanical Factors Explain Everything?
Source: PLoS One. 2013 Apr 3;8(4):e60491. doi: 10.1371/journal.pone.0060491 (PMC3616143; doi:10.1371/journal.pone.0060491)
Supplement: Text S1 — CoM location in obese: using an Additional Mass Index and picture segmentation to adjust Winter's anthropometric table. (DOCX) [file pone.0060491.s002.docx]

**CoM location in obese: using an Additional Mass Index and picture segmentation to adjust Winter's anthropometric table**

The location of the CoM and its monitoring during the movement cannot be directly performed from motion analysis systems. The CoM, barycenter of all body segments, is a mobile virtual point in an intra- or extra-body space and is dependent on the body-segmental geometry. One of the most commonly used techniques in biomechanical studies is a calculation using the Winter’s [1] geometric anthropometric proportional table. **Figure S1a** shows the main anatomical landmarks and CoM segmental positioning corresponding to a healthy subject, according to the Winter's table. However, this table is only applicable for healthy adults, e.g., non obese participants in the present study. Using it on atypical or pathological morphologies, such as obese subjects, requires some specific adjustments in order to take into account the individual anthropometric characteristics.

The CoM estimation is dependent on two variables: (1) the mass of each body segment relative to the total body mass and (2) the position of the CoM of each body segment relative to the distance separating the two anatomical landmarks characterizing this segment. In order to adapt Winter's anthropometric table for obese subjects and thus to better estimate CoM location, we introduced an Additional Mass Index (AMI) to first correct the mass of each body segment and we used trunk profile photographs to adjust the CoM of the trunk segment.

Adjustment of the mass of the body segments in obese subjects

The Body Mass Index (BMI), expressed in kg.m^-^² is calculated as :

$BMI=\frac{M}{H^{2}}$, where *M* it the total body mass in kg and *H* the height of the subject in m. We consider the optimal BMI as the median value of the normal BMI range [18.5 – 25 kg.m^-^²] for a male subject: *BMI_opt_* = 21.75 kg.m^-2^

For a male subject of given height *H*, one can calculate an optimal body mass *M_opt_* to reach the optimal *BMI_opt_*: $M_{opt}=BMI_{opt}.H^{2}$

Consider now an obese subject of given height *H* and overweight mass *M*, the AMI (δ) is defined by $M=M_{opt}+\delta M_{opt}$. Note that $\frac{\delta}{1+\delta}$ is the percentage of mass to loose in order to reach *BMI_opt_.*

*M_opt_* can be directly expressed as a function of δ and *M*: $M_{opt}=\frac{1}{1+\delta}M$. The BMI can also be expressed relatively to *BMI_opt_*:$BMI=\frac{M}{H^{2}}=\frac{M_{opt}+\delta M_{opt}}{H^{2}}=(1+\delta).BMI_{opt}$.

The present method consists in considering an obese subject of mass *M* relatively to an optimal weight subject of equal height *H* but of mass *M_opt_* . In order to compute the mass of each body segment from the Winter's table, we estimate the distribution of $\delta M_{opt}$, the difference of mass between the obese and the optimal weight subject, on the different body segments. Lets consider $\Delta m^{s}$ the additional mass of a given segment *s*, i.e., the proportion of $\delta M_{opt}$ that can be attributed to *s* when *s* is part of an overweight body. We consider also $k_{w}^{s}$ the relative mass of segment s compared to the whole body mass as given in the Winter's anthropometric tables, i.e., relative to an optimal weight subject. Some authors suggested that the increase in head, neck, hands and feet mass in obese subjects is negligible compare to the excess of weight [2-3], therefore$\Delta m^{head}=\Delta m^{neck}=\Delta m^{hand}=\Delta m{}^{feet}=0$. Assuming that the distribution of $\delta M_{opt}$ applied to the legs, thighs, arms and forearms segments is approximately proportional to the distribution of the segmental masses reported by Winter, we can compute $\Delta m^{ll}$ (lower limbs without the feet) and $\Delta m^{ul}$ (upper limbs without the hands):

$\Delta m^{ll}=\delta M_{opt}.2(k_{w}^{leg}+k_{w}^{thigh})=\delta M_{opt}.2(0.0446+0.1)=0.2892\delta M_{opt}$ and $\Delta m^{ul}=\delta M_{opt}.2(k_{w}^{forearm}+k_{w}^{arm})=\delta M_{opt}.2(0.016+0.028)=0.088\delta M_{opt}$

Consequently, the trunk will receive all the residual mass not distributed in $\Delta m^{ll}$ or in $\Delta m^{ul}$ : $\Delta m^{trunk}=\delta M_{opt}-\Delta m^{ll}-\Delta m^{ul}=0.6228\delta M_{opt}$

The additional mass relatively to the optimal mass is distributed for approximately 30% to the lower limbs, 10% to the upper limbs and 60% to the trunk.

As $k_{w}^{trunk}=0.497$, the total mass of the trunk of an obese subject is $M^{trunk}=k_{w}^{trunk}M_{opt}+\Delta m^{trunk}=\frac{0.497+0.6228\delta}{1+\delta}M$

Applying this formula for the median type 2 obese of our study (BMI = 36.6 Kg.m^-^²), we have: $\delta=0.682$, $M^{trunk}=54.8\%M$ (the mass of the trunk segment represents approximately 55% of the total mass).

Considering the additional charge of 20% of initial body weight applied in control experiment #1, we have:$M^{trunk}=69.7\%M$ which corresponds to a higher relative weight of the trunk.

Estimation of the trunk segment CoM in obese subjects

Estimation of CoM position of the trunk segment can be improved by using a profile photograph of the trunk and by individually estimate this location with respect to the hip and shoulder markers. The trunk contour was semi-automatically segmented by dividing the trunk in 20 rectangular area between the anatomical landmarks of the hip (greater trochanter) and shoulder (acromion) (see **Figure S1b**). Even if the software allowed the selection of a specific body density for each area, we deliberately chosen to apply a constant density of 1 in all areas, as a study suggested that the estimate of the inertial body is more sensitive to geometrical than to densitometric factors which have ultimately little impact on the overall result [4]. Once the AMI, $M^{trunk}$, and trunk CoM location are correctly estimated, they can be used in the classical equation for calculating the whole body CoM: $CoM=\frac{1}{M}\sum M^{s}\times CoM^{s}$

A statistical comparison of this adjustment method with the conventional method (without adjustment) was performed to control the benefits of the present appendix work. We compared anteroposterior and vertical CoM displacement during the descending movement, analyzed with these two methods and for six trials from six obese participants, randomly selected. The statistical analysis revealed a significant difference between the two methods. For the A-P CoM displacement (% of anat BoS), the result was 132.9 ± 30 vs. 154.4 ± 3.27 without and with adjustment, respectively, t test, t = -4.07, p = 0.0096). For the vertical CoM displacement (% of anat BH), the result was (26.6 ± 3.1 vs. 28.63 ± 3.4 without and with adjustment, respectively, t test = -2.78, p = 0.039). The six trials used for this comparison are illustrated in **Figure S1c**.

**Reference**

1. Winter DA (1990) Biomechanics and motor control of human movement. Whiley, New York.
2. Chowdhury B, Kvist H, Andersson B, Bjorntorp P, Sjostrom L (1994) CT-determined changes in adipose tissue distribution during a small weight reduction in obese males. Int. J. Obesity Related Metab. Disord. 17: 685–691.
3. Kotani K, Tokunaga K, Fujioka S, Kobatake T, Keno Y, et al. (1994) Sexual dimorphism of age-related changes in whole-body fat distribution in the obese. Int J Obes Relat Metab Disord. 18: 207-202.
4. Wicke J, Dumas GA (2010) Influence of the volume and density functions within geometric models for estimating trunk inertial parameters. J Appl Biomech 26: 26-31.
